# COVID-19 vaccination acceptance among Syrian population: a nationwide cross-sectional study

**DOI:** 10.1186/s12889-021-12186-6

**Published:** 2021-11-18

**Authors:** Mosa Shibani, Mhd Amin Alzabibi, Abd El-Fattah Mouhandes, Tamim Alsuliman, Angie Mouki, Hlma Ismail, Shahd Alhayk, Ahmad Abdulateef Rmman, Marah Mansour, Marah Marrawi, Nawras Alhalabi, Mhd Baraa Habib, Mhd Kutaiba Albuni, Ahmad Al-Moujahed, Bisher Sawaf, Data Collection Group, Hanaa Wael Zahrawi, Anan Mhd tawfik Bakdounes, Ahmad Nabil Alhouri, Nour Zuhir Abdulal, Rama Adnan Hawari, Mhd Homam Safiah, Sara Omar Alazrak, Rama Ahmad Alsayed Husein, Mohammed Moutaz Alshaghel, Wafik Mayo, Abdallah Aladna, Abdulkader Hajjar Mwaffak, Shahd Maarrawi, Marah Khalil, Lana Almahairi, Yaman Mokresh, Mouhammad Saleh Younes

**Affiliations:** 1 Faculty of Medicine, Syrian Private University, Mazzeh Street, P.O. Box 36822, Damascus, Syrian Arab Republic; 2 Faculty of Medicine, University of Aleppo, Aleppo, Syria; 3 Hematology and Cell Therapy Department, Saint-Antoine Hospital, AP-HP Sorbonne University, Paris, France; 4 Faculty of Pharmacy, Maykop State Technological University, Maykop, Russia; 5 Faculty of Medicine, Tartous university, Tartous, Syria; 6 Department of Statistics, Syrian Private University, Damascus, Syria; 7 Department of Ophthalmology, Faculty of Medicine, Damascus University, Damascus, Syria; 8 Department of Internal Medicine, Hamad Medical Corporation, Doha, Qatar; 9 Department of Ophthalmology, Byers Eye Institute, Stanford University, Palo Alto, CA USA

**Keywords:** COVID-19, Vaccine, Acceptance, SARS-CoV-2, Syria

## Abstract

**Background:**

Severe Acute Respiratory Syndrome Coronavirus 2 continues to spread globally and in the absence of an effective treatment, the vaccine remains the best hope for controlling this disease. In this study, we seek to find out the extent to which people in Syria accept the Corona vaccine and what are the factors that affect their decision.

**Methods:**

This cross-sectional study was conducted in Syria during the period from January 3 to March 17, 2021. A structured self-administered questionnaire was distributed in two phases: The first phase included distributing the questionnaire as a Google Form on social media platforms. In the second phase, a paper version of the questionnaire was handed to patients, their companions, and workers in public hospitals. SPSS v.25 and R v.4.1.1 were used to analyze the data. Pearson Chi-square test and Logistic Regression were used to study the associations between categorical groups.

**Results:**

Of 7531 respondents, 3505 (46.5%) were males and 4026 (53.5%) were females. 3124 (41.5%) were 18–24 years old. Healthcare workers were participants’ main sources of information (50.9%), followed by Social Media users (46.3%). 2790 (37%) of the participant are willing to be vaccinated, and 2334 (31%) were uncertain about it. Fear of possible side effects was the main reason for the reluctance to take the vaccine 1615 (62.4%), followed by mistrust of the vaccine formula 1522 (58.8%). 2218 (29.5%) participants think COVID-19 poses a major risk to them personally. Vaccination intention was significantly associated with gender, residence, financial status, educational level, and geographic origin.

**Conclusion:**

This study showed very negatively important results. The study participants Vaccination acceptance rate is almost the lowest when compared to its peers. A Lot of efforts should be made to correct misinformation about the vaccine and answer all questions about it, especially with a health system that has been ravaged by war for 10 years.

**Supplementary Information:**

The online version contains supplementary material available at 10.1186/s12889-021-12186-6.

## Background

Coronavirus disease 2019 (COVID-19) has rapidly become a global threat, affecting (as of December 2019) over 173 million people, causing approximately 4 million deaths, and continuing to affect all aspects of life worldwide [1]. Despite applying various preventive measures to minimize the spread of severe acute respiratory syndrome coronavirus 2 (SARS-CoV-2), in the absence of effective treatment, immunization may be presented as the best hope for containing the disease. As of February 18th, 2021, at least seven different vaccines have been rolled out in various countries, and 102 more vaccines are undergoing clinical trials [2]. The vaccine will play a vital role in reducing infections even among the unvaccinated population, through herd immunity. However, to attain herd immunity, the development phase should be followed by a large-scale vaccination program. Based on the current data, (60–75%) of the population should yet acquire the immunity to achieve this endpoint [3].

To the date of writing, 24,700 laboratory-confirmed cases and 1799 casualties of COVID-19 have been recorded by the Syrian Ministry of Health [4]. However, official numbers are relatively low, probably due to the limited numbers of tests and their relatively high cost.

Ten years of war in Syria have overburdened the population and created multiple economic, social, and educational difficulties. As a result of this widespread war, the country’s economy is shattered, and the healthcare system is on the verge of collapse [5]. In the light of the recent inflation, the number of People in Need (PIN) is expected to increase from 11 million in 2020 to 13.3 million in 2021 [5]. The majority of Syrian families cannot afford self-isolation and physical distancing, due to the huge rise in personal protective equipment, and medical supplies prices alongside other COVID-19 related factors [6]. The aforementioned situation highlights the extreme importance of vaccine availability, as it may present the only effective way to limit the spread of SARS-CoV-2 in this war-torn country.

On February 25th, 2021, the Syrian Ministry of Health announced the start of vaccine administration to frontline and healthcare workers (HCWs). And set up a health platform dedicated for the public to register for vaccination on May 5th, 2021.

However, the number and source of these vaccines remain ambiguous. Noteworthy, Syria is eligible for COVID-19 vaccines (COVAX) under the COVAX Facility’s Advance Market Commitment. It received its first batch of the AstraZeneca vaccine in April 2021 (1,020,000 doses dedicated for 3% of the population and targeted high-risk groups) [6].

The behavior and willingness for vaccination are one of the success-determining factors for disease contentment and re-normalization of daily life [7, 8]. According to the Strategic Advisory Group of Experts on Immunization (SAGE), vaccine hesitancy is the unwillingness or reluctance to take the vaccine despite its availability [9]. In 2019, the World Health Organization (WHO) has listed vaccine hesitancy as one of the top 10 threats to global health [10]. While vaccine hesitancy has been an ongoing public health issue for years, the rapid development of vaccines in response to the COVID-19 pandemic has exacerbated the issue. The ongoing infodemic, the rarity of reliable Arabophone sources of information, and the lack of a strict educational policy may accentuate the mediocre situation in Syria [11].

In this study, we aim to measure the willingness of the Syrian population to receive the COVID-19 vaccine, report their beliefs about the vaccine, and study the variables that affect their decision.

## Methods

### Study design, setting, and participants

A cross-sectional survey was conducted aiming at the general population in all major cities in Syria (Damascus, Rif Dimashq, Aleppo, Daraa, Deir ez-Zor, Hama, Al-Hasakah, Qamishli, Homs, Idlib, Latakia, Quneitra, Ar-Raqqah, As-Suwayda, Tartous). The data were collected during the period from January 3 to March 17, 2021.

A nationally representative sample of Syrian people was questioned using a structured self-administered questionnaire which was developed based on previously existing literature, and then it underwent some modification to be suitable for the Syrian society [12–14]. The questionnaire was then piloted on 15 persons to insure its clarity and adjustments were made based on their feedback. Because of the war that has been going on in Syria for ten years, many residents do not have access to the Internet either because the necessary infrastructure has been destroyed or because of the bad economic situation. Therefore, to ensure the correct diversity in the sample and to avoid selection bias, we distributed the questionnaire in two phases: The first phase ran from January 3 to February 1, 2021, in which the questionnaire was distributed as Google Form on social media platforms (Facebook, Whatsapp, and Twitter). The second phase ran from February 2 to March 17 and included the distribution of the questionnaire in person, in which the questionnaire was handed to patients, their companions, and workers in public hospitals in each of Damascus, Homs, Aleppo, Tartous, Hama, and Sweida governorates. During this period in which the questionnaire was distributed, the daily level of confirmed COVID-19 cases in Syria was at a constant or declining rate, and vaccines were not available in the country yet, which would ensure - to a reasonable degree - that the respondents’ opinion was not affected by the time of their response to the questionnaire. Chain-referral sampling was used in online distribution phase, online respondents were asked to send the questionnaire to their family members, friends, and colleagues. Convenience sampling methods were used in the hard copy distribution phase.

The sample size was calculated using Openepi online software available at “https://www.openepi.com/SampleSize/SSPropor.htm”. According to data from United Nations, the estimated number of Syrian population in 2019 is about 18 million [15], based on that, we conducted a statistical power analysis for sample size calculation. With a conservative assumption of 50% vaccine acceptance, an alpha of 0.05 and a power of 80% and a desired margin of error of 1.14%, the desired sample size was found to be 7336. Inclusion criteria included that the person is: (1) 18 years old or more, (2) literate, (3) Syrian (or equivalent) and living in Syria, (4) willing to complete the questionnaire. Of the 10,083 participants who responded to the questionnaire, 551 were excluded for not meeting the inclusion criteria (817 have withdrawn their consent to participate, 1043 were not Syrians or Syrians living outside Syria, and 692 did not answered all the questions). This gave us a final sample size of (7531) participants.

### Measures

The questionnaire consisted of 32 questions divided into 3 sections:

1. *Socio-demographic characteristics*: This covers 14 questions about age [as 4 age groups (18–24, 25–44, 45–65, and >  65) years], gender, marital status, nationality, the governorate of origin [the governorates were divided into 5 categories based on geographical location 1- Central governorates (Damascus, Rif Dimashq, Hama, Homs), 2- Eastern governorates (Deir ez-Zor, Al-Hasakah, Ar-Raqqah), 3- Western governorates (Latakia, Tartous), 4- Northern governorates (Aleppo, Idlib), 5-Southern governorates (Daraa, Quneitra, As-Suwayda)], place of residence (urban or rural), financial status, employment status, educational level, father’s and mother’s educational level, having health insurance, working or studying in a healthcare-related field.
2. *Beliefs and opinions about COVID-19 and a COVID-19 vaccination:* This section includes 4 questions about the previous infection with SARS-CoV-2 and about the risk this virus poses on the person and on the Syrian society as a whole, and 11 statements regarding general beliefs and opinions about the COVID-19 vaccine
3. *Willingness to take the vaccine.* This section consisted of 3 questions. First, to measure vaccination willingness we asked respondents to report how probably they would be to take the COVID-19 vaccine when it is accessible for them on an eleven-point scale from 0 to 10 (0 = very unlikely, 10 = very likely), then the answers were categorised into three groups: very Likely (8, 9, 10), Uncertain (3, 4, 5, 6, 7), and very Unlikely (0, 1,2) this scoring system was used in previously published study in the United Kingdom (UK) [12]. Second, asking about whether free vaccination will affect the decision on taking it. And finally, the reason(s) for not taking the vaccine.

### Statistical analysis

Data from the hard copy questionnaires was entered manually by the investigators to the original Google Form online questionnaire that used to collect online data, then t data was extracted from Google Form directly to an Excel spreadsheet. After that, we encoded the raw data in the Excel sheet to make it compatible with the statistics software.

We used Statistical Package for Social Sciences version 25.0 (SPSS Inc., Chicago, IL, United States) to perform Chi-square univariate analysis, and R v 4.1.1 to conduct Logistic Regression model. Categorical variables were reported as frequencies and percentages. Pearson Chi-square test was used to study the associations between categorical groups. *p* value < 0.05 was considered statistically significant.

### Ethical consideration

The study protocol was approved by the Research Ethics Committee in each of Damascus, Aleppo, Tartous, and Syrian Private Universities, and the ethical committees in the concerned hospitals. Informed consent was obtained from every participant prior to participation.

## Results

### Participant’s characteristics

Of 10,083 participant, 7531 agreed to participate and fully completed the questionnaire completely (response rate = 74.7%), of which 3505 (46.5%) were males and 4026 (53.5%) were females. Regarding the age groups, 3124 (41.5%) were 18–24 years old, 2338 (31%) were 25–44 years old, 1686 (22.4%) were 45–65 years old, and 383 (5.1%) were above the age of 65 years. The majority of the participants were city residents 5711 (75.8%). University students 2906 (38.6%) and University graduates 1908 (25.3%) represented the majority of responders, while those who did not receive formal education represented only 324 (4.3%). When asked if they work or study in a healthcare-related field, 4936 (65.5%) of the participants answered (No). The vast majority of the respondents 6042 (80.2%) do not have health insurance. Geographically, 4376 (58.1%) were from central governorates, 1272 (16.9%) were from northern governorates, 1165 (15.5%) were from western governorates, 365 (4.8%) were from eastern governorates, and 353 (4.7%) were from Southern governorates. Regarding financial status, 3241 (43%) reported their status as [Average (covers only the basics of life)], 2651 (35.2%) as [Good (covers the basics and some extras)], 1268 (16.8%) as [Bad (doesn’t even cover the basics of life)], and only 371 (4.9%) reported their status as [Excellent (covers all basics and many luxuries)]. Of the participants, 2653 (35.2%) were students, and 1688 (22.4%), 1114 (14.8%), 1635 (21.7%) were working a full-time job, working a part-time job, or unemployed, respectively. Regarding the respondents’ sources of information, Healthcare workers were the most chosen source (50.9%), followed by Social Media (Facebook, WhatsApp, YouTube, Telegram, etc.) (46.3%), and Local and international health organizations (Syrian Ministry of Health, WHO, etc.) and Scientific journals (43.5%) (Table 1).

**Table 1:** Participant’s Characteristics

Variables		Total (%) *n* = 7531
Age range (years)	18–24	3124 (41.5%)
	25–44	2338 (31%)
	45–65	1686 (22.4%)
	> 65	383 (5.1%)
Gender	Male	3505 (46.5%)
	Female	4026 (53.5%)
Marital status	Single	3984 (52.9%)
	Married	2825 (37.5%)
	In relationship	500 (6.6%)
	Widow	222 (2.9%)
Do you have health insurance?	Yes	1489 (19.8%)
	No	6042 (80.2%)
Educational level	No formal education	324 (4.3%)
	Elementary school	422 (5.6%)
	Secondary school	550 (7.3%)
	Highschool	782 (10.4%)
	University student	2906 (38.6%)
	University graduate	1908 (25.3%)
	Postgraduate degree	639 (8.5%)
Do you work or study in the healthcare system	Yes	2595 (34.5%)
	No	4936 (65.5%)
Mother’s educational level	No formal education	1225 (16.3%)
	Primary school	1834 (24.4%)
	Secondary school	1656 (22%)
	University degree	2625 (34.9%)
	Postgraduate degree	191 (2.5%)
Residency	City	5711 (75.8%)
	Countryside	1820 (24.2%)
Geographical origin	Eastern Syria	365 (4.8%)
	Northern Syria	1272 (16.9%)
	Middle Syria	4376 (58.1%)
	Southern Syria	353 (4.7%)
	Western Syria	1165 (15.5%)
Financial status	Bad	1268 (16.8%)
	Middle	3241 (43%)
	Good	2651 (35.2%)
	Excellent	371 (4.9%)
Employment	Unemployed	1635 (21.7%)
	Part-time job	1114 (14.8%)
	Full-time job	1688 (22.4%)
	Student	2653 (35.2%)
	Student + Part-time job	290 (3.9%)
	Student + Full-time job	131 (1.7%)
	Part-time + Full-time jobs	20 (0.3%)
Father’s educational level	No formal education	760 (10.1%)
	Primary school	1971 (26.2%)
	Secondary school	1512 (20.1%)
	University degree	2776 (36.9%)
	Postgraduate degree	512 (6.8%)
What is the source(s) of your information regarding COVID-19 pandemic?	common conversation and words of mouth.	(22.6%)
	Healthcare workers.	(50.9%)
	Media and advertising (TV, Radio, Ads, Newspapers, etc).	(40.2%)
	Local and international health organizations (Syrian Ministry of Health, WHO, etc) and Scientific journals.	(43.5%)
	Social Media (Facebook, Youtube, etc).	(46.3%)

### Beliefs and opinions about COVID-19 and a COVID-19 vaccination

When asked “Do you think you have contracted Coronavirus?”, over half of the participants 4549 (60.4%) answered “No”, 2742 (36.4%) answered “Yes, I had the Symptoms”, and just 240 (3.2%) answered “Yes, and confirmed with Polymerase Chain Reaction (PCR)”. Moreover, 4571 (60.7%) stated that they personally knew someone (excluding themselves) who has had PCR-confirmed coronavirus disease. 4639 (61.6%) of the study population believe that COVID-19 poses a major risk to people in Syria, whereas only 2218 (29.5%) of them think it poses the same risk to them personally. (Table 2).

**Table 2:** Participants’ beliefs and risk perceptions regarding COVID-19

		Total (%), *n* = 7531
Do you believe you have had coronavirus?	No	4549 (60.4%)
	yes with PCR	240 (3.2%)
	Yes with symptoms	2742 (36.4%)
Do you personally know anyone (excluding yourself) who has had coronavirus and confirmed with PCR?	No	2960 (39.3)
	Yes	4571 (60.7)
To what extent do you think coronavirus poses a risk to people in Syria?	No risk at all	314 (4.2%)
	Minor risk	1621 (21.5%)
	Major risk	4639 (61.6%)
	Do not know	956 (12.7%)
To what extent do you think coronavirus poses a risk to you personally?	No risk at all	834 (11.1%)
	Minor risk	3224 (42.8%)
	Major risk	2218 (29.5%)
	Do not know	1255 (16.7%)

2771 (36.8%) of participants agreed that the vaccine is just a way for manufacturing companies to earn money and 5509 (73.2%) stated that they will be worried from experiencing side effects from vaccination. Regarding whether or not the vaccine should be mandatory for everyone who is able to have it, 3230 (42.9%) said it should not. Half of the participants 3787 (50.3%) are not confident that the vaccine will protect them against the disease. 4984 (66.2%) believe that they do not have enough information about the vaccine to make an informed decision about whether or not to get vaccinated. (Table 3).

**Table 3:** Participant’s beliefs regarding COVID-19 vaccine

opinions toward the vaccination	Agree (N%)	Disagree (N%)	DNK (N%)
The vaccine is just a way for manufacturing companies to earn money.	2771 (36.8%)	1865 (24.8%)	2895 (38.4%)
I would be worried about experiencing side effects from a coronavirus vaccination	5509 (73.2%)	1079 (14.3%)	943 (12.5%)
A coronavirus vaccine will allow us to get back to ‘normal’	2694 (35.8%)	1512 (20.1%)	3325 (44.2%)
A coronavirus vaccination should be mandatory for everyone who is able to have it	3140 (41.7%)	3230 (42.9%)	1161 (15.4%)
If I get a coronavirus vaccination, I will be protected against coronavirus	1918 (25.5%)	1826 (24.2%)	3787 (50.3%)
A coronavirus vaccination could give me coronavirus	1713 (22.7%)	2495 (33.1%)	3323 (44.1%)
A coronavirus vaccination will be too new for me to be confident about getting vaccinated	4821 (64%)	1162 (15.4%)	1548 (20.6%)
I know enough about the coronavirus illness to make an informed decision about whether or not to get vaccinated	4136 (54.9%)	1544 (20.5%)	1851 (24.6%)
I know enough about the coronavirus vaccine to make an informed decision about whether or not to get vaccinated	2547 (33.8%)	2320 (30.8%)	2664 (35.4%)
If a coronavirus vaccination were recommended by the Government, I would get vaccinated	3755 (49.9%)	2092 (27.8%)	1684 (22.4%)
If a coronavirus vaccination were recommended by a healthcare professional, I would get vaccinated	4967 (66%)	1309 (17.4%)	1255 (16.7%)

### Willingness to take the vaccine

**Fig. 1:**
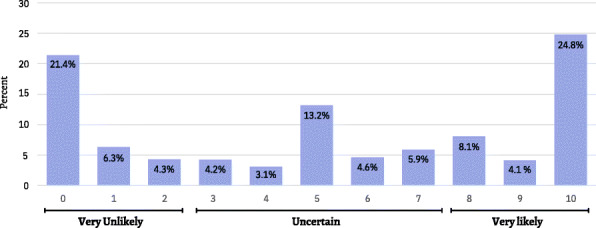
When the vaccine become available to you, how likely is it that you will have one?

**Fig. 2:**
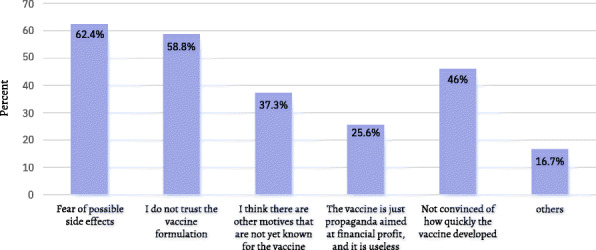
If the vaccine is available for free, what are the reasons to not have one?

When asked “on a scale from 0 to 10 (0 = very unlikely, 10 = very likely) when a coronavirus vaccination becomes available to you, how likely is it you will have one?”, 2407 (32%) of the participants answered [very Unlikely (0 + 1 + 2)], 2334 (31%) answered [Uncertain (3 + 4 + 5 + 6 + 7)], and 2790 (37%) answered [very Likely (8 + 9 + 10)] (Fig. 1). Furthermore, 2226 (29.6%) said that they will take the vaccine only if it is free, 2718 (36.1%) stated they will take it regardless if it is free or not. When the group of respondents who answered that they will never take the vaccine were asked about the reasons for their decision, the most frequent answer was “Fear of possible side effects” 1615 (62.4%), followed by “I do not trust the vaccine formulation” 1522 (58.8%), “I do not have confidence in the rapid development of the vaccine” 1189 (46%) and, “I think there are other motives that are not yet known for the vaccine” 966 (37.3%). (Fig. 2).

### Correlations between vaccination intention and participants characteristics

In the Chi-square univariate analysis, the results showed a statistical significance difference between males and females in willingness to get the vaccine, as males were more likely to get it (43.1% vs. 31.8%, × 2 = 105.190, *P*-value < 0.0001). A significant association was found between the age groups and intentions to get vaccinated, where participants in the 45–65 years old group were more likely to get vaccinated than others (40.2%, × 2 = 109.792, *P*-value < 0.0001). Married participants (40.2%, × 2 = 109.792, *P*-value < 0.0001) and participants from Eastern governorates of Syria (40.2%, × 2 = 109.792, *P*-value < 0.0001) were more likely to get a vaccine. The intentions to get a vaccine were significantly associated with residency and financial status with participants resident in rural areas (42.3% vs. 35.4%, × 2 = 29.372, *P*-value < 0.0001) and those in bad financial status (40.9%, × 2 = 32.017, *P*-value < 0.0001) more likely to get vaccinated. Surprisingly, participants with lower educational levels (no education: 52.8%, elementary school: 54.3%) were most likely to have a vaccine (× 2 = 189.934, *P*-value < 0.0001). (Table 4).

**Table 4:** Correlation between vaccination intentions and participants’ characteristics

Characteristic variables		When the vaccine becomes available to you, how likely is it that you will have one?			Chi-Square value	*P*-value
		Unlikely	Uncertain	Likely		
Age	18–24 (*n* = 3124)	851 (27.2%)	1154 (27.2%)	1119 (35.8%)	109.792	< 0.0001
	25–44 (*n* = 2338)	829 (35.5%)	(28.5%)	843 (36.1%)		
	45–65 (*n* = 1686)	591 (35.1%)	418 (24.8%)	677 (40.2%)		
	> (*n* = 383)	136 (35.5%)	96 (25.1%)	151 (39.4%)		
sex	Male (*n* = 3505)	989 (28.2%)	1005 (28.7%)	1511 (43.1%)	105.190	< 0.0001
	Female (*n* = 4026)	1418 (35.2%)	1329 (33.0%)	1279 (31.8%)		
marital status	Single (*n* = 3984)	1162 (29.2%)	1395 (35.0%)	1427 (35.8%)	89.243	< 0.0001
	Married (*n* = 2825)	1009 (35.7%)	710 (25.1%)	1106 (39.2%)		
	in relationship (*n* = 500)	154 (30.8%)	176 (35.2%)	170 (34.0%)		
	Widow (*n* = 222)	82 (36.9%)	53 (23.9%)	87 (39.2%)		
Origin	Eastern Syria (*n* = 365)	94 (25.8%)	104 (28.5%)	167 (45.8%)	41.834	< 0.0001
	Northern Syria (*n* = 1272)	356 (28%)	380 (29.9%)	536 (42.1%)		
	Middle Syria (*n* = 4376)	1476 (33.7%)	1346 (30.8%)	1554 (35.5%)		
	Southern Syria (*n* = 353)	122 (34.6%)	107 (30.3%)	124 (35.1%)		
	Western Syria (*n* = 1165)	359 (30.8%)	397 (34.1%)	409 (35.1%)		
Residency	City (*n* = 5711)	1860 (32.6%)	1831 (32.1%)	2020 (35.4%)	29.372	< 0.0001
	Countryside (*n* = 1820)	547 (30.1%)	503 (27.6%)	770 (42.3%)		
Financial status	Bad (*n* = 1268)	434 (34.2%)	315 (24.8%)	519 (40.9%)	32.017	< 0.0001
	Middle (*n* = 3241)	1019 (31.4%)	1035 (31.9%)	1187 (36.6%)		
	Good (*n* = 2654)	844 (31.8%)	874 (33.0%)	933 (35.2%)		
	Excellent (*n* = 371)	110 (29.6%)	110 (29.6%)	151 (40.7%)		
Employment	Unemployed (*n* = 1635)	575 (35.2%)	439 (26.9%)	621 (38.0%)	99.068	< 0.0001
	part-time (*n* = 1114)	405 (36.4%)	336 (30.2%)	373 (33.5%)		
	full- (*n* = 1688)	587 (34.8%)	445 (26.4%)	656 (38.9%)		
	Student (*n* = 2653)	715 (27.0%)	979 (36.9%)	959 (36.1%)		
	part-time + student (*n* = 290)	83 (28.6%)	91 (31.4%)	116 (40.0%)		
	full-time + student (*n* = 131)	38 (29.0%)	38 (29.0%)	55 (42.0%)		
	part-time + full-time (*n* = 20)	4 (20.0%)	6 (30.0%)	10 (50.0%)		
Academic level	no education (*n* = 324)	87 (26.9%)	66 (20.4%)	171 (52.8%)	189.934	< 0.0001
	Elementary (*n* = 422)	101 (23.9%)	92 (21.8%)	229 (54.3%)		
	Secondary (*n* = 550)	204 (37.1%)	150 (27.3%)	196 (35.6%)		
	Highschool (*n* = 782)	301 (38.5%)	228 (29.2%)	253 (32.4%)		
	university student (*n* = 2906)	803 (27.6%)	1057 (36.4%)	1046 (36.0%)		
	university graduate (*n* = 1908)	719 (37.7%)	551 (28.9%)	638 (33.4%)		
	post-university study (*n* = 639)	192 (30.0%)	190 (29.7%)	(40.2%)		
Father’s educational level	no education (*n* = 760)	209 (27.5%)	154 (20.3%)	397 (52.2%)	120.106	< 0.0001
	primary education (*n* = 1971)	704 (35.7%)	615 (31.2%)	652 (33.1%)		
	secondary education (*n* = 1521)	523 (34.6%)	462 (30.6%)	527 (34.9%)		
	university degree (*n* = 2776)	822 (29.6%)	948 (34.1%)	1006 (36.2%)		
	post-uni (*n* = 512)	149 (29.1%)	155 (30.3%)	208 (40.6%)		
Mother’s educational level	no education (*n* = 1225)	355 (29.0%)	268 (21.9%)	602 (49.1%)	144.687	< 0.0001
	primary education (*n* = 1834)	660 (36.0%)	567 (30.9%)	607 (33.1%)		
	secondary education (*n* = 1656)	579 (35.0%)	532 (32.1%)	545 (32.9%)		
	university degree (*n* = 2625)	763 (29.1%)	917 (34.9%)	945 (36.0%)		
	post-uni (191)	50 (26.2%)	50 (26.2%)	91 (47.6%)		

Respondents who thought that COVID-19 poses a major risk to them personally were more willing to take the vaccine (50.9%, × 2 = 336.178, *P*-value < 0.0001). (Table 5). However, Logistic Regression model revealed that only gender, geographical origin, residency, employment, and educational level are significantly correlated with intention to get the vaccine (*P* < 0.0001). (Supplementary file 1).

**Table 5:** Correlation between vaccination intentions and risk perceptions regarding COVID-19

		When the vaccine becomes available to you, how likely is it that you will have one?			Chi-Square test	
		Unlikelyn (%)	Uncertainn (%)	Likelyn (%)	Chi-Square	p.value
To what extent do you think coronavirus poses a risk to people in Syria?	No risk at all (*n* = 314)	112 (35.7%)	50 (15.9%)	152 (48.4%)	399.056	< 0.0001
	Minor risk (*n* = 1621)	766 (47.3%)	469 (28.9%)	386 (23.8%)		
	Major risk (*n* = 4640)	1175 (25.3%)	1458 (31.4%)	2007 (43.3%)		
	Do not know (*n* = 956)	354 (37.0%)	357 (37.3%)	245 (25.6%)		
Total (*n* = 7531)		2407 (32%)	2334 (31%)	2790 (37%)		
To what extent do you think coronavirus poses a risk to you personally?	No risk at all (*n* = 834)	386 (46.3%)	190 (22.8%)	258 (30.9%)	336.178	< 0.0001
	Minor (*n* = 3224)	(32.9%)	1143 (35.5%)	1020 (31.6%)		
	Major risk (*n* = 2218)	550 (24.8%)	540 (24.3%)	1128 (50.9%)		
	Do not know (*n* = 1255)	410 (32.7%)	461 (36.7%)	384 (30.6%)		
Total (*n* = 7531)		2407 (32%)	2334 (31%)	2790 (37%)		

## Discussion

To our best of knowledge, this is the first nationally representative study conducted in Syria regarding vaccination intentions.

Although availability, effectiveness, and cost of the vaccine are key factors to determine the success of a vaccine, vaccine hesitancy plays a crucial role too [16, 17]. Only 37% of our population answered: “very likely” when asked whether they will take the vaccine upon its availability or not and 31% answered “uncertain”. These numbers raise a lot of concern, especially with the fact that immunization of at least 60–75% of individuals is required to achieve desired herd immunity [3]. Our results are much lower than other studies in United States of America (57.6%), Canada (80%), Libya (79.6%), Saudi Arabia (64.7%), Lebanon (58.8%), Iraq (61.7%), Qatar (42.7%), China (91.3%), Italy (53.7%), Poland (56.3%), Denmark (80%), Germany (70%), Portugal (75%), Russia (54.9%), and United Kingdom (55.8%). But higher than those in Kuwait (23.6%) and Jordan (28.4%) [8, 12, 13, 18–26].

Canada has the biggest per-capita hoard of the vaccines reserved, enough vaccines to immunize its citizens five times. The US and UK came second [27]. Out Of 832 million vaccine doses, only 0.2% have been sent to low-income countries [28, 29]. Unfair vaccination rollouts may indicate a deeper issue of inequality and jeopardize the health of fragile populations -such as Syria-, which has enough doses to immunize only 4% of the population [30].

With the fragile and weak health care system, vaccination offers the best hope for Syrians to fight against COVID-19. Appreciating this, about one-third (36.1%) are willing to pay to get vaccinated. Higher numbers were observed in other low-income countries like Libya (48.2%) and Lebanon (51.9%) [18, 20].

The official number of confirmed cases in Syria is to the date of writing 24,700 cases [4]. However, (60.7%) of our participants said that they personally knew at least someone who has had PCR-confirmed coronavirus disease, and (36.4%) said that they experienced COVID-19 symptoms, which strongly suggests that the community transmission of the disease surpasses the official numbers.

Interestingly, an optimism bias was observed among our study population, since most of them believe themselves to be less at risk of COVID-19 than others in the society [31]. This may be attributed to the fact that Syria has a relatively youthful community [32], and younger people tend to think they are at less risk to be infected. A similar observation was reported in the UK, France, and Lebanon [12, 20, 33].

In our study, only 4136 (54.9%) considered themselves knowledgeable enough about COVID-19 to make an informed decision about vaccination, this is relatively lower than the previous two studies that measured the knowledge of the Syrian population through structured questionnaires, which showed a mean knowledge ranging from 60 to 75.6% [34, 35]. This indicates that the Syrian population underestimates their knowledge and does not trust the knowledge they have. This disparity can be attributed to the fact that many participants rely on unreliable sources of information, as almost half of the participants get their information from social media. Social media information may be very misleading, especially in low income countries [11]. It can be anti-vaccine oriented as reported in several previous studies [36–39]. Moreover, only (33.8%) thought they have enough information about the vaccine, this can also be attributed to their reliance on unreliable sources, which will affect their quality of information and hence, their intentions to be vaccinated. Furthermore, unreliable sources of information will also help in the spread of conspiracy theories about the vaccine and this was evident in our results as (37.3%) of the participants think there are hidden motives behind the vaccine. Our results are similar to the numbers reported in Libya (38.7%), but much higher than the Lebanese study (19.7%). A US study showed that those who believed conspiracies are 3.9 times less likely to be vaccinated [40]. An educational framework must be set to counter those conspiracies and their effect on vaccination and the spread of COVID-19. Although the most common side effects of the vaccine are mild and resolve after 1–2 days such as mild fever, injection site reaction and fatigue [41], most of the participants in our study were reluctant to take the vaccine due to fear of its side effects. This may be a result of the media’s focus on the rare few cases in which some people have experienced serious and life-threatening side effects. Our results are similar to those reported in Qatar, Lebanon, UK, and Libya [12, 18, 20, 22]. The second most common reason for not taking the vaccine in our study is the lack of confidence in the vaccine formulation. Perhaps because of newly introduced technologies, as it is the first time people learned about vaccines that use Messenger RNA was with the Pfizer-Biontech vaccine [42]. The remarkable speed with which the Corona vaccine was developed made it more likely to be questioned by the public, which is evident in our study, as 42% of the participants said that they were not convinced of the speed of developing the vaccine. These results are in agreement with other studies in Lebanon and Australia [20, 43]. It should be noted that the types of vaccines that would be available in Syria were not yet known at the time of the distribution of this questionnaire. The type of vaccine may play a role in the behavior of vaccine hesitancy, especially since some countries have some preferences among the types of vaccines available. This may make some people -students who want to travel to their home countries- reluctant to take one of the types of vaccines even if it is available.

## Conclusion

Our findings reveal alarming levels of vaccine acceptability among the Syrian population. This indicates that there is a knowledge gap regarding many aspects of the vaccine and the disease. Targeted nationwide campaigns aimed at raising awareness of the population about the developing process, side effects, and formulation of the vaccine are needed to make the people more confident in the vaccine and aware of its importance in controlling this pandemic and eventually achieve herd immunity. Furthermore, The population must be educated about using reliable sources of information and not relying on other people or social media as a source of information to avoid fallacies that would contribute to the continued spread of the pandemic. The economic and humanitarian situation in war-torn Syria requires all concerned health organizations to take serious actions to secure sufficient numbers of vaccines to cover most of the Syrian community as soon as possible.

### Strengths and limitations

The demographic distribution in our study is fairly representative of Syrian society according to data from the Central Bureau of Statistics (CBS), Damascus, Syria. On its latest report, CBS reported that ~ 40% of the Syrian population were below 24 years old, 25.5% were 25–44 years old, 17.6% were 45–65 years old, and 5.3% were above 65 years old [44].

The study has some limitations. First, the cross-sectional study type may not be able to infer causality and association; thus, additional longitudinal studies are required to reach more accurate results. Second, using an online distribution method makes it harder to reach all classes of Syrian society, especially lower socioeconomic classes and older ages; we managed this limitation by distributing the questionnaire in two stages, where the second stage included its distribution in paper form on the ground.

Third, this study was conducted in a country that suffers from two major crises, with very specific circumstances, this may limit the ability to generalize the results only to countries with a similar situation. Finally, the vaccine acceptance rate of the population in our study may not reflect a true acceptance of the vaccine in reality. Especially since at the time of distributing the questionnaire, Syria had not received any doses of the vaccine, and therefore, the results should be interpreted with caution.

## Supplementary Information

**Additional file 1.**

## Data Availability

The datasets used and/or analyzed during the current study are available from the corresponding author on reasonable request.
