# Supplementary material for: COVID-19 vaccination acceptance among Syrian population: a nationwide cross-sectional study
Source: BMC Public Health. 2021 Nov 18;21:2117. doi: 10.1186/s12889-021-12186-6 (PMC8598277; doi:10.1186/s12889-021-12186-6)
Supplement: Supplementary file 1 — Additional file 1. [file 12889_2021_12186_MOESM1_ESM.pdf]

Supplementary file. 1

Results of the full logistic regression model analysing association between participants characteristics and vaccination intention

```
knitr::opts_chunk$set(echo = TRUE)
```

```
setwd("E:/Projects/Mousa")
```

```
data_sheet_edited<-read.csv('Data_sheet_edited.csv', header = TRUE, sep = ',')
```

```
modelvvv<- glm(Vaccine~sex+origin+residency+employment+academic_level, data = data_sheet_edited)
summary(modelvvv)
```

```
##
## Call:
## glm(formula = Vaccine ~ sex + origin + residency + employment +
##     academic_level, data = data_sheet_edited)
##
## Deviance Residuals:
##      Min        1Q    Median        3Q       Max
## -1.42667  -0.92994  -0.00851   0.85317   1.21989
##
## Coefficients:
##                 Estimate Std. Error t value Pr(>|t|)
## (Intercept)     1.225134   0.032926  37.209  < 2e-16 ***
## sex            -0.164524   0.019141  -8.595  < 2e-16 ***
## origin         -0.031941   0.009699  -3.293 0.000995 ***
## residency       0.089345   0.022598   3.954 7.77e-05 ***
## employment      0.039761   0.007637   5.206 1.98e-07 ***
## academic_level -0.030548   0.006944  -4.399 1.10e-05 ***
## ---
## Signif. codes:  0 '***' 0.001 '**' 0.01 '*' 0.05 '.' 0.1 ' ' 1
##
## (Dispersion parameter for gaussian family taken to be 0.6737768)
##
##     Null deviance: 5177.5  on 7530  degrees of freedom
## Residual deviance: 5070.2  on 7525  degrees of freedom
## AIC: 18406
##
## Number of Fisher Scoring iterations: 2
```

```
modelvf<-glm(Vaccine~age+sex+marital_status+origin+residency+financial_status+
           employment+academic_level+father_educational+mother_educational, data = data_sheet_ed
ited)
summary(modelvf)
```

```
##
## Call:
## glm(formula = Vaccine ~ age + sex + marital_status + origin +
##     residency + financial_status + employment + academic_level +
##     father_educational + mother_educational, data = data_sheet_edited)
##
## Deviance Residuals:
##      Min        1Q    Median        3Q       Max
## -1.39551  -0.91852  -0.01745   0.86414   1.26441
##
## Coefficients:
##                     Estimate Std. Error t value Pr(>|t|)
## (Intercept)         1.269095   0.046752  27.145  < 2e-16 ***
## age                -0.023546   0.014534  -1.620 0.105260
## sex                -0.178502   0.019746  -9.040  < 2e-16 ***
## marital_status     -0.002188   0.014274  -0.153 0.878154
## origin             -0.033442   0.009880  -3.385 0.000716 ***
## residency           0.095947   0.023014   4.169 3.09e-05 ***
## financial_status    0.020588   0.013990   1.472 0.141173
## employment          0.023226   0.009494   2.446 0.014451 *
## academic_level     -0.042184   0.007925  -5.323 1.05e-07 ***
## father_educational  0.020488   0.012600   1.626 0.103996
## mother_educational -0.002308   0.012662  -0.182 0.855336
## ---
## Signif. codes:  0 '***' 0.001 '**' 0.01 '*' 0.05 '.' 0.1 ' ' 1
##
## (Dispersion parameter for gaussian family taken to be 0.6731052)
##
##     Null deviance: 5177.5  on 7530  degrees of freedom
## Residual deviance: 5061.8  on 7520  degrees of freedom
## AIC: 18404
##
## Number of Fisher Scoring iterations: 2
```
